# Risk Factors for Hypothalamic Obesity in Patients With Adult-Onset Craniopharyngioma: A Consecutive Series of 120 Cases

**DOI:** 10.3389/fendo.2021.694213

**Published:** 2021-07-28

**Authors:** Wei Wu, Quanya Sun, Xiaoming Zhu, Boni Xiang, Qiongyue Zhang, Qing Miao, Yongfei Wang, Yiming Li, Hongying Ye

**Affiliations:** ^1^Department of Endocrinology and Metabolism, Huashan Hospital, Fudan University, Shanghai, China; ^2^Department of Neurosurgery, Huashan Hospital, Fudan University, Shanghai, China

**Keywords:** craniopharyngioma, adult-onset, weight gain, hypothalamic obesity, hypothalamus involvement

## Abstract

**Context:**

Hypothalamic obesity (HO) is a severe complication following craniopharyngioma, but studies regarding the sequelae in adult-onset patients with craniopharyngioma are sparse.

**Objective:**

The objective of the study was to describe weight changes after surgical treatment in adult-onset craniopharyngioma patients and to analyze risk factors for postoperative weight gain and HO.

**Subjects and Method:**

A retrospective analysis was conducted of 120 adult-onset patients who underwent surgery for craniopharyngioma and follow-up at the institution of the authors between January 2018 and September 2020. Clinical characteristics, anthropometric data, image features, treatment modalities, and endocrine indices were collected. Multivariable logistic regression analysis was used to identify independent risk factors for postoperative weight gain and HO.

**Results:**

Forty-nine (40.8%) patients had clinically meaningful weight gain (≥5%) in a median follow-up time of 12.0 months (range 1.0–41.0 months) after surgery. The mean postoperative weight gain in this subgroup was 17.59 ± 12.28 (%). Weight gain continued in the first year following surgery. Patients with lower preoperative BMI [OR 0.78, 95% CI (0.67–0.90), P = 0.001] and the adamantinomatous subtype [OR 3.46, 95% CI (1.02–11.76), P = 0.047] were more likely to experience postoperative weight gain ≥5%. The prevalence of HO was 19.2% preoperatively and increased to 29.2% at last follow-up postoperatively. Only preoperative BMI [OR 2.51, 95% CI (1.64–3.85), P < 0.001] was identified as an independent risk factor for postoperative HO.

**Conclusions:**

HO is a common complication in patients with adult-onset craniopharyngioma. Patients with higher preoperative BMI had a greater risk for developing HO postoperatively.

## Introduction

Craniopharyngiomas are embryological tumors arising from the remnants of Rathke’s pouch along the craniopharyngeal canal. They are mainly located in the sellar/parasellar region, accounting for 2–5% of all the primary intracranial neoplasms (1). They can be detected at any age, and a bimodal age distribution has been reported, with one peak during 5–9 years in children and another during 55–69 years in adults (1). Although histologically benign, the tumor often grows aggressively, causing damage to surrounding vital structures, such as the optic apparatus, the pituitary gland, and the hypothalamus. The quality of life of craniopharyngioma survivors is greatly impaired by substantial long-term morbidities, including endocrinopathies, visual defects, hypothalamic damage, and impaired cognitive function (2–4). Another common and troubling morbidity is HO, which indicates rapid and dramatic weight gain in patients with tumors or lesions in the hypothalamic region. HO not only increases risks for metabolic and cardiovascular diseases, but also contributes to excess morbidity and mortality (5, 6).

Previous studies showed 32–70% of child-onset patients with craniopharyngioma developed HO after surgical treatment (7–9). However, far less is known about postoperative weight change in patients with adult-onset craniopharyngioma. In three previous studies (10–12) that comprise both childhood-onset and adult-onset craniopharyngioma patients, rates of HO vary from 41.5 to 67% in adult-onset group, but exact weight change after surgery is unknown. Moreover, although several risk factors for HO were identified in the entire cohort, such as age under 10 years old, initial symptoms of intracranial hypertension, pterional surgery, and recurrence (12), no correlation analysis was carried out separately in adult-onset patients. In a series of 28 adult-onset patients with craniopharyngioma, Dr. Van Gompel described a significant postoperative weight gain and found a positive correlation between the degree of hypothalamic involvement and postoperative weight gain (13). Since all of the above studies were carried out in Caucasian patients, the postoperative weight change and risk factors for HO in Asian adult-onset craniopharyngioma patients remain unknown.

In this study, we performed a retrospective analysis of Chinese adult-onset craniopharyngioma patients to assess postoperative weight change and to determine independent risk factors for HO.

## Methods

### Subjects

Subjects were recruited from all craniopharyngioma patients admitted to the endocrine department of Huashan hospital (Shanghai, China), one of the largest pituitary centers in China, between January 2018 and September 2020, a period during which regular follow-up was suggested to all the patients with craniopharyngioma following surgery. The recommended follow-up time points were before surgery and 1, 3, 6, 12 months after surgery, then every year for the subsequent 5 years. Anthropometric measurements, including height and body weight, and assessment of hypothalamic–pituitary function were performed at each follow-up in clinic or inpatient department, which was preferred by the patient. Contrast enhanced magnetic resonance imaging (MRI) of the sellar region was taken during 3 to 6 months following operation, then yearly in the subsequent 5 years. If any symptoms or signs indicating relapse of the tumor occurred, MRI and reevaluation of the pituitary function were suggested immediately. Patients were eligible for this study if: 1) diagnosed at age of 18 years or more; 2) pathologically confirmed craniopharyngioma; 3) surgery and at least one follow-up performed at Huashan hospital; 4) receiving sufficient hormone replacement except for growth hormone therapy.

**Figure 1:**
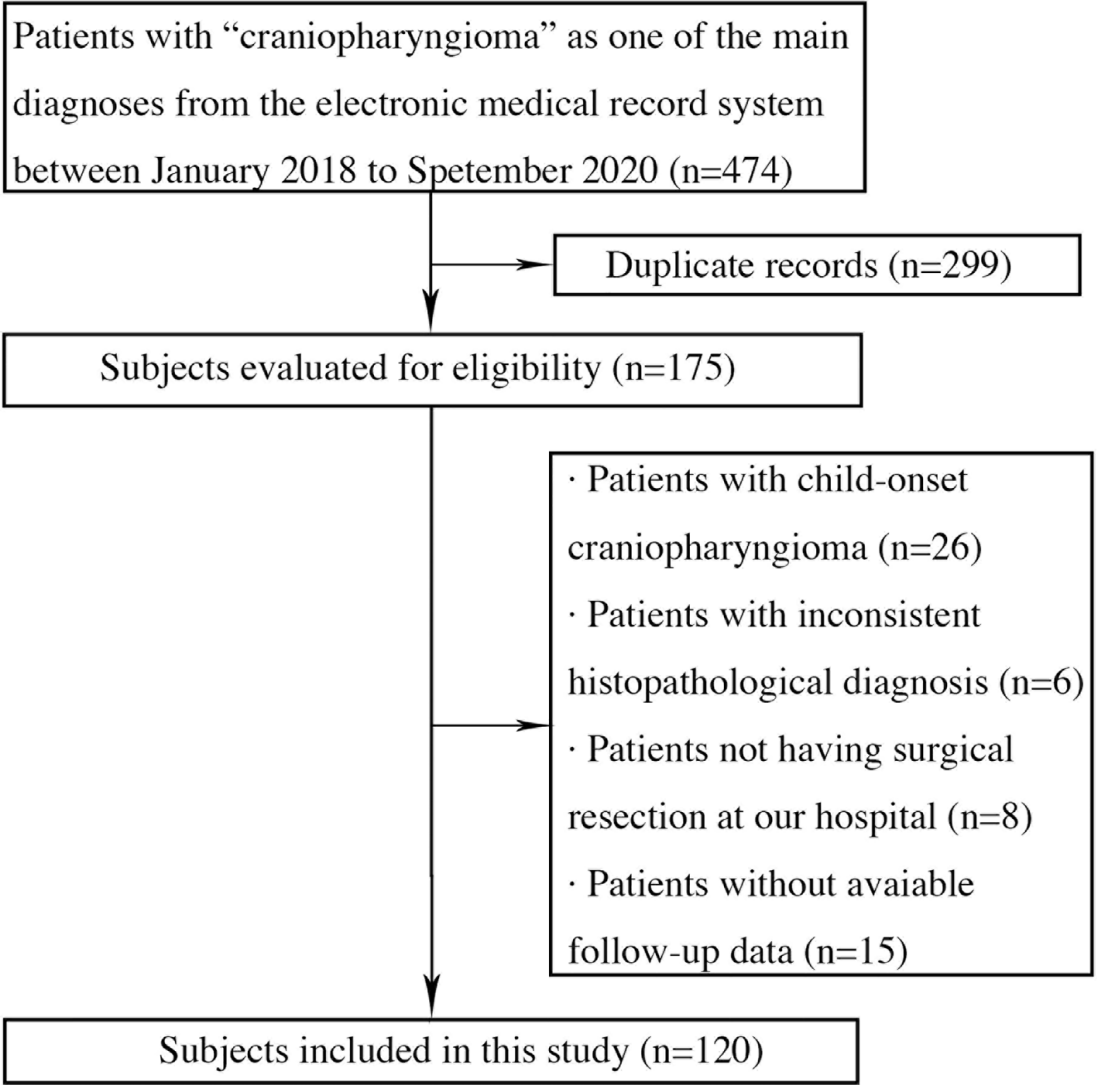
Flow chart of subjects included in this study.

A sum of 474 records were retrieved from our electronic medical record system using the term “craniopharyngioma” as one of the main diagnoses, among which 299 were duplicate records. In the remaining 175 records, 26 patients were excluded for child-onset craniopharyngioma, 6 for inconsistent histopathological diagnosis, 8 for not having surgical resection at our hospital and 15 for without available follow-up data. Ultimately, a total of 120 cases of adult-onset craniopharyngioma patients were included, and a retrospective analysis was performed. A detailed follow chart of inclusion and exclusion of patients was shown in **Figure 1**. All subjects were informed of the purpose of this study and signed a written consent form. This study was approved by the Institutional review board of Huashan Hospital, Fudan University.

### Clinical Data and Definitions

Medical records of each patient were reviewed, and a spreadsheet was designed for the collection of demographic information, anthropometric measurements, image characteristics, treatment modalities, pathologic subtypes, and endocrine functions.

The data of weight and height at diagnosis and at follow-up were used for analysis. Height was measured using a ruler to the nearest centimeter, and weight was measured on a digital scale wearing lightweight clothing to the nearest 0.1 kg. BMI was calculated using the following formula: BMI = weight (kg)/height (m^2^). Overweight was defined as BMI between 24 and 28 kg/m^2^, and obese with BMI ≥28 kg/m^2^, according to the Working Group on Obesity in China (14). Postoperative weight changes were calculated as follows: [weight at follow-up (kg) − preoperative weight (kg)]/preoperative weight (kg) × 100%. Consistent with previous studies (15, 16), postoperative weight changes ≥5% were considered a clinically meaningful weight gain.

Neuroradiologic characteristics were identified from preoperative MRI or computed tomography of the sellar region in all cases. Tumor size was recorded using the maximum tumor diameter. Tumor consistencies were divided into predominantly cystic, predominantly solid and mixed. Preoperative and postoperative hypothalamus involvement was classified into three categories using the grading system reported by Puget (17). Extent of surgery was estimated based on postoperative MRI.

Pituitary function was evaluated preoperatively and at regular follow-up postoperatively. Central adrenal insufficiency was defined by basal serum cortisol level <3 μg/dl measured at 8 a.m. or peak cortisol level <18.1 μg/dl after insulin tolerance test or ACTH stimulation test. Central hypothyroidism was diagnosed based on free T4 level below the reference range combined with a low or normal TSH. In premenopausal women, central hypogonadism was defined by oligomenorrhea or amenorrhea combined with low serum estradiol and inappropriately low or normal FSH and LH levels. Gonadotrophin deficiency in postmenopausal women was diagnosed by serum FSH and LH within premenopausal range. In men, central hypogonadism was defined as low serum testosterone in conjunction with low gonadotropins. Serum IGF-1 level was measured, although insulin tolerance test was not performed routinely to detect growth hormone deficiency in this study population. Clinical presentation, urine-specific gravity, urine and serum osmolality, serum sodium level, and the need for desmopressin treatment were comprehensively evaluated for the diagnosis of central diabetes insipidus (CDI). Water deprivation testing was also performed if necessary. Moreover, all patients with pituitary deficits were placed on appropriate hormone replacement except for GH.

### Statistical Analysis

Continuous variables were described with means ± SDs, medians and interquartile ranges as appropriate. Categorical variables were expressed as frequency and percentage. Comparisons of continuous variables between two independent groups were performed using the Student’s t-test or Mann–Whitney U test. Chi-square tests or Fisher exact tests were used to compare categorical variables. Paired t-test was used for preoperative and postoperative body weight and BMI. Wilcoxon signed-rank tests were used to compare pre- and postoperative pituitary deficit number and IGF-1 level. Comparisons of the ratios of preoperative and postoperative pituitary deficits were performed using the McNemar’s test. Multivariable logistic regression was performed to identify independent risk factors for postoperative weight gain ≥5% and HO. We compared demographic characteristics, anthropometric measurements, image features, treatment modalities, pathologic subtypes, and endocrine functions between postoperative weight gain <5% and ≥5% groups or between postoperative HO and non-HO groups. Next, parameters with a P-value <0.10 were included in multivariable logistic regression analyses adjusting for confounders. Cofounders were chosen based on clinical experiences and what were reported as potential risk factors for weight gain or HO. Hosmer and Lemeshow test was conducted to assess the goodness of fit of the regression models. Overall predictive accuracies of these models were also recorded. Predictive accuracies and P-values of Hosmer and Lemeshow tests of multivariable logistic regression models were shown in **Supplemental Table S1**. Receiver operating characteristic (ROC) curve was used to find the best cut-off value of preoperative BMI for predicting postoperative HO. All analyses were performed using SPSS (version 20.0, SPSS Institute, Chicago, IL) and Prism 5.0 for Windows (GraphPad Software, Inc.), and a two-sided level of P <0.05 was considered statistically significant.

## Results

### Clinical Features

Clinical features of all patients are displayed in **Table 1**. Mean age at diagnosis was 42.58 ± 13.67 years. The ratio of male *vs*. female was 1.4:1. The median duration of follow-up time was 12.0 months (range 1.0–41.0 months). There were 48.3% of patients having intracranial hypertension symptoms, such as headache and vomiting. The average tumor size was 30.11 ± 9.52 mm, and nearly one half of the tumors had a combination of solid and cystic components. In addition, 45% of patients had grade 2 hypothalamus involvement based on Puget’s classification preoperatively, with 37.5% of patients having grade 1 hypothalamus involvement and 17.5% having no hypothalamus involvement. Endoscopic endonasal approach was the predominant choice in our study; only seven patients underwent transcranial approach, such as pterional and supraorbital eyebrow approaches. Gross total resection was achieved in 84 (70.0%) patients. Radiotherapy was used either preoperatively or postoperatively as an adjuvant treatment in 15 (12.5%) patients, including conventional fractionated external beam irradiation, gamma knife, and cyber knife. Pathological subtypes were available in a majority of patients. The ratios of adamantinomatous variant *vs*. papillary variant were 69:32. Hypothalamus involvement was reassessed postoperatively based on Puget’s grading system. More patients (38.3%) had no hypothalamus involvement, with 35.0% of patients having grade 1 hypothalamus involvement and 26.7% having grade 2 hypothalamus involvement. During the follow-up period, tumor relapse was observed in 29 (24.2%) patients, who then received repeated surgeries or radiotherapy. No one died during this study.

**Table 1:** General characteristics of 120 adult patients with craniopharyngioma, 2018–2020.

Variables	Total (N = 120)	Postoperative weight gain≥5% (N = 49)	Postoperative weight gain<5% (N = 71)	P-value	Postoperative HO group (N = 35)	Postoperative non-HO group (N = 85)	P-value
Age at onset, years	40.82±13.81	38.06±14.25	42.72±13.26	0.069	39.22±12.59	41.47±14.30	0.421
Age at diagnosis, years	42.58±13.67	39.08±14.01	44.99±12.98	0.019	40.57±12.43	43.40±14.13	0.305
Gender, male (%)	70 (58.3%)	28 (57.1%)	42 (59.2%)	0.826	24 (68.6%)	46 (54.1%)	0.144
Follow-up time, months	12.0 (4.0-14.0)	12.0 (4.0–15.5)	12.0 (4.0–13.0)	0.460	12.0 (6.0–16.0)	12.0 (4.0–13.0)	0.115
Intracranial hypertension symptoms	58 (48.3%)	24 (49.0%)	34 (47.9%)	0.906	20 (57.1%)	38 (44.7%)	0.215
Preoperative body weight, kg	68.76±13.71	64.31±11.66	71.83±14.25	0.003	80.77±14.24	63.81±9.97	<0.001
Preoperative BMI, kg/m^2^	24.50±4.12	22.75±3.06	25.70±4.34	<0.001	28.60±4.16	22.81±2.68	<0.001
Body weight at last follow-up, kg	72.70±14.00	75.42±14.40	70.82±13.50	0.077	87.79±12.01	66.48±9.23	<0.001
BMI at last follow-up, kg/m^2^	25.90±4.10	26.69±4.07	25.35±4.06	0.080	31.03±2.45	23.78±2.42	<0.001
Image characteristics							
Tumor size, mm	30.11±9.52	31.53±9.64	29.13±9.38	0.182	31.33±8.28	29.63±9.97	0.386
Tumor consistency				0.649			0.278
Predominantly cystic, n (%)	38 (31.7%)	14 (28.6%)	24 (33.8%)		9 (25.7%)	29 (34.1%)	
Predominantly solid, n (%)	27 (22.5%)	13 (26.5%)	14 (19.7%)		6 (17.1%)	21 (24.7%)	
Mixed, n (%)	55 (45.8%)	22 (44.9%)	33 (46.5%)		20 (57.1%)	35 (41.2%)	
Preoperative hypothalamus involvement, n (%)				0.178			0.001
Grade 0	21 (17.5%)	10 (20.4%)	11 (15.5%)		4 (11.4%)	17 (20.0%)	
Grade 1	45 (37.5%)	12 (24.5%)	29 (40.8%)		6 (17.1%)	39 (45.9%)	
Grade 2	54 (45.0%)	27 (55.1%)	31 (43.7%)		25 (71.4%)	29 (34.1%)	
Postoperative hypothalamus involvement, n (%)				0.351			0.596
Grade 0	46 (38.3%)	19 (38.8%)	27 (38.0%)		11 (31.4%)	35 (41.2%)	
Grade 1	42 (35.0%)	14 (28.6%)	28 (39.4%)		14 (40.0%)	28 (32.9%)	
Grade 2	32 (26.7%)	16 (32.7%)	16 (22.5%)		10 (28.6%)	22 (25.9%)	
Treatment parameters							
Surgical approach				1.000			0.695
Endoscopic endonasal approach	113 (94.2%)	46 (93.9%)	67 (94.4%)		32 (91.4%)	81 (95.3%)	
Transcranial approach	7 (5.8%)	3 (6.1%)	4 (5.6%)		3 (8.6%)	4 (4.7%)	
Extent of surgery				0.321			0.707
Gross total resection	84 (70.0%)	38 (77.6%)	46 (64.8%)		26 (74.3%)	58 (68.2%)	
Subtotal resection	30 (25.0%)	9 (18.4%)	21 (29.6%)		7 (20.0%)	23 (27.1%)	
Partial resection	6 (5.0%)	2 (4.1%)	4 (5.6%)		2 (5.7%)	4 (4.7%)	
Radiotherapy, n (%)	15 (12.5%)	2 (4.1%)	13 (18.3%)	0.021	5 (14.3%)	10 (11.8%)	0.939
Pathologic subtype^*^				0.062			0.703
Adamantinomatous variant, n (%)	69 (68.3%)	33 (78.6%)	36 (61.0%)		22 (71.0%)	47 (67.1%)	
Papillary variant, n (%)	32 (31.7%)	9 (21.4%)	23 (39.0%)		9 (29.0%)	23 (32.9%)	
Tumor relapse, n (%)	29 (24.2%)	10 (20.4%)	19 (26.8%)	0.424	9 (25.7%)	20 (23.5%)	0.799

^*^Pathologic subtypes of nineteen patients are missing.

Prevalence of preoperative and postoperative endocrine deficiencies was shown in **Tables 2** and **3**. Both hypopituitarism of each axis and central diabetes insipidus (28.3% preoperatively *vs*. 81.7% postoperatively, P < 0.001) were much more frequent after surgery. The most common anterior pituitary deficits were hypogonadism (77.5% preoperatively *vs*. 97.1% postoperatively, P = 0.003), followed by hypothyroidism (45.0% preoperatively *vs*. 87.5% postoperatively, P < 0.001) and adrenal insufficiency (35.8% preoperatively *vs*. 85% postoperatively, P < 0.001). The median number of pituitary deficits increased from 2.0 (1.0–3.0) preoperatively to 4.0 (3.0–4.0) postoperatively. In addition, there were remarkably more patients having four pituitary deficits at last follow-up (15.8% preoperatively *vs*. 74.2% postoperatively, P < 0.001). Although growth hormone deficiencies were not assessed routinely in this study, serum IGF-1 was markedly decreased postoperatively (131.50 (99.83–175.00) preoperatively *vs*. 91.90 (63.90–127.00) postoperatively, μg/L, P < 0.001). Likewise, the rate of IGF-1 below the age and gender specific reference range greatly increased from 37.5% preoperatively to 85% postoperatively (P < 0.001).

**Table 2:** Preoperative pituitary function.

	Total (N = 120)	Postoperative weight gain ≥5% (N = 49)	Postoperative weight gain <5% (N = 71)	P-value	Postoperative HO group (N = 35)	Postoperative non-HO group (N = 85)	P-value
Adrenal insufficiency	43 (35.8%)	20 (40.8%)	23 (32.4%)	0.344	13 (37.1%)	30 (35.3%)	0.848
Hypothyroidism	54 (45.0%)	23 (46.9%)	31 (43.7%)	0.723	14 (40.0%)	40 (47.1%)	0.480
Hypogonadism	93 (77.5%)	38 (77.6%)	55 (77.5%)	0.991	30 (85.7%)	63 (74.1%)	0.167
Central diabetes insipidus	34 (28.3%)	18 (36.7%)	16 (22.5%)	0.090	12 (34.3%)	22 (25.9%)	0.353
Number of pituitary deficits	2.0 (1.0-3.0)	2.0 (1.0-3.0)	1.0 (1.0-3.0)	0.333	2.0 (1.0-3.0)	2.0 (1.0-3.0)	0.531
Four pituitary deficits	19 (15.8%)	10 (20.4%)	9 (12.7%)	0.254	6 (17.1%)	13 (15.3%)	0.801
IGF-1 (μg/L)	131.50 (99.83–175.00)	132.50 (103.30–182.50)	130.50 (98.75–169.50)	0.724	146.50 (113.50–187.75)	124.50 (85.73–169.50)	0.081
IGF-1 below the reference range	45 (37.5%)	22 (44.9%)	23 (32.4%)	0.164	12 (34.3%)	33 (38.8%)	0.641

**Table 3:** Postoperative pituitary function.

	Total (N = 120)	Postoperative weight gain ≥5% (N = 49)	Postoperative weight gain <5% (N = 71)	P-value	Postoperative HO group (N = 35)	Postoperative non-HO group (N = 85)	P-value
Adrenal insufficiency	102 (85.0%)	41 (83.7%)	61 (85.9%)	0.735	31 (88.6%)	71 (83.5%)	0.482
Hypothyroidism	105 (87.5%)	44 (89.8%)	61 (85.9%)	0.528	31 (88.6%)	74 (87.1%)	0.820
Hypogonadism	108 (90.0%)	45 (91.8%)	63 (88.7%)	0.804	34 (97.1%)	74 (87.1%)	0.181
Central diabetes insipidus	98 (81.7%)	43 (87.8%)	55 (77.5%)	0.152	32 (91.4%)	66 (77.6%)	0.076
Number of pituitary deficits	4.0 (3.0–4.0)	4.0 (3.5–4.0)	4.0 (3.0–4.0)	0.672	4.0 (4.0–4.0)	4.0 (3.0–4.0)	0.159
Four pituitary deficits	89 (74.2%)	37 (75.5%)	52 (73.2%)	0.780	29 (82.9%)	60 (70.6%)	0.163
IGF-1 (μg/L)	91.90 (63.90–127.00)	91.40 (54.68–113.75)	92.40 (65.80–128.00)	0.519	93.10 (60.20–107.00)	91.65 (65.28–133.75)	0.445
IGF-1 below the reference range	102 (85.0%)	43 (87.8%)	59 (83.1%)	0.483	31 (88.6%)	71 (83.5%)	0.482

### Postoperative Weight and BMI

**Figure 2:**
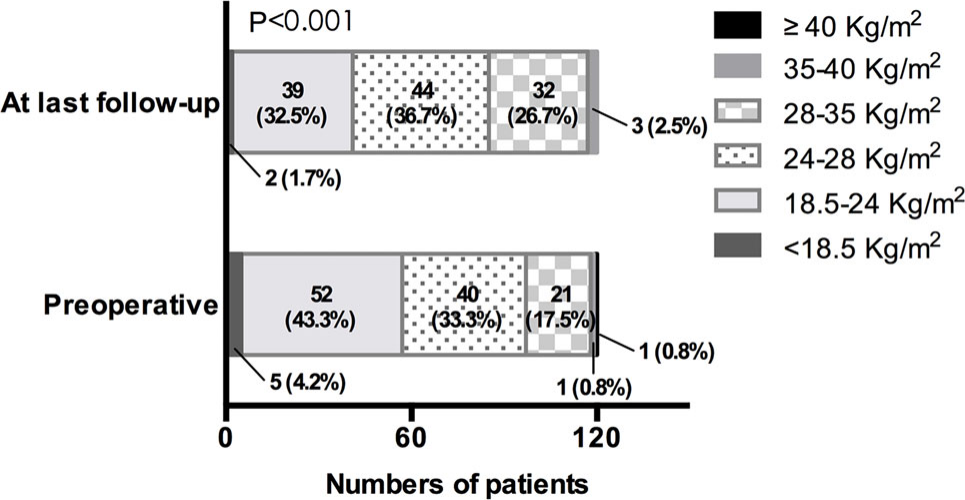
BMI distribution preoperatively and at last follow-up. BMI of <18.5 kg/m^2^ for low body weight, BMI of 18.5–24.0 kg/m^2^ for normal body weight, BMI of 24.0–28.0 kg/m^2^ for overweight, BMI of 28.0–35.0 kg/m^2^ for Class I obesity, BMI of 35.0–40.0 kg/m^2^ for Class II obesity, BMI of ≥40.0 kg/m^2^ for Class III obesity.

**Figure 3:**
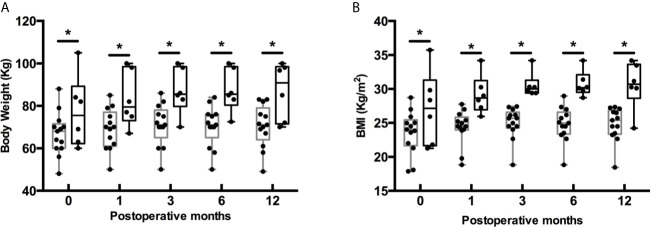
Postoperative body weight **(A)** and BMI **(B)** at follow-up in a subset of patients who finished all the four follow-up time points. Gray box indicates patients without postoperative HO (n = 13), and black box indicates patients with postoperative HO (n = 6). Boxes depict the 95% confidence interval. Horizontal lines indicate medians. Whiskers depict the minimum and maximum data. Dots represent each individual measurement. *P < 0.05.

In our cohort, the mean preoperative body weight and BMI were 68.76 ± 13.71 kg and 24.50 ± 4.12 kg/m^2^ separately (**Table 1**). At last follow-up after surgery, the mean body weight and BMI markedly increased to 72.70 ± 14.00 kg (P < 0.001) and 25.90 ± 4.10 kg/m^2^ (P<0.001). Similarly, there were a higher proportion of overweight and obese patients at last follow-up (P < 0.001) (**Figure 2**). To investigate the pattern of postoperative weight gain, we analyzed the body weight and BMI at each follow-up time points. As shown in **Table 4**, the completion rates of each follow-up visit were 78/120 (65.0%), 80/120 (66.7%), 60/120 (50.0%), 67/114 (58.8%), 19/42 (45.2%), and 5/15 (33.3%) for 1, 3, 6, 12, 24, and 36 months following operation separately. Both body weight and BMI kept rising within the first year after surgery. In a small subgroup of patients (n = 19) who have finished all the four follow-up time points within the first 12 months following surgery, we found patients with postoperative HO had significant higher body weight and BMI than those without postoperative HO not only at baseline, but also at each follow-up time points (**Figures 3A, B** **)**. Interestingly, on a visit at 24 months after surgery, remarkable higher body weight and BMI were noticed in this cohort, which returned to the level of 3 months after operation on a visit at 36 months (**Table 4**). However, since only a third of patients have met the 24 month time point and an even smaller part of subjects have met the 36 month time point at the end of the study, explanations to these observations should be with caution. Further studies with longer follow-up period are needed to unravel the trend of postoperative weight change after 1 year.

**Table 4:** Body weight and BMI at each follow-up.

Months after surgery	N^*^	Body weight (kg)	BMI (kg/m^2^)
1 month	78/120	71.15±12.03	25.38±3.65
3 months	80/120	72.53±13.22	25.68±3.99
6 months	60/120	73.82±14.77	26.27±4.32
12 months	67/114	74.11±13.84	26.22±3.80
24 months	19/42	82.15±15.02	28.57±3.99
36 months	5/15	72.10±14.72	25.47±4.05

^*^The numerator represents the number of patients with available data; the denominator stands for the number of patients who met the follow-up time point.

### Risk Factors for Postoperative Weight Gain ≥5%

At last follow-up, 49 (40.8%) patients had weight gain ≥5%. The mean postoperative weight gain was 17.59 ± 12.28 (%) in this category, while 71 (59.2%) patients kept their body weight constant or experienced a minor weight loss. Clinical features based on these two postoperative weight gain categories were shown in **Table 1**. Age at diagnosis was younger in the postoperative weight gain ≥5% group (39.08 ± 14.01 *vs*. 44.99 ± 12.98, P = 0.019). In addition, preoperative body weight (64.31 ± 11.66 *vs*. 71.83 ± 14.25kg, P = 0.003) and BMI (22.75 ± 3.06 *vs*. 25.70 ± 4.34kg/m^2^, P < 0.001) were much lower in the postoperative weight gain ≥ 5% group. Yet patients with postoperative weight gain≥5% tended to have higher body weight (75.42 ± 14.40 *vs*. 70.82 ± 13.50kg, P = 0.077) and BMI (26.69 ± 4.07 *vs*. 25.35 ± 4.06kg/m^2^, P = 0.080) at last follow-up, although statistical significance was not reached. There was a lower percentage of patients who undertook radiotherapy in the postoperative weight gain ≥5% group (4.1 *vs*. 18.3%, P=0.021). The pathologic subtypes seemed to be different between these two groups, with a higher percentage of patients with adamantinomatous variant in the postoperative weight gain ≥5% group (78.6 *vs*. 61.0%, P = 0.062). No differences were found in the ratio of males, follow-up months, percentages of patients with intracranial hypertension symptoms, image characteristics, surgical treatment parameters, and the rate of tumor relapse between the two groups. As shown in **Table 2**, preoperative pituitary function was comparable between the two groups, except that patients with postoperative weight gain ≥5% had a higher percentage of CDI preoperatively (36.7 *vs*. 22.5%, P = 0.090) although not statistically significant. Similarly, no differences were found in the postoperative pituitary function between the two groups (**Table 3**).

We further performed multivariable logistic regression analysis to identify independent risk factors for postoperative weight gain ≥5% (**Table 5**). Age at diagnosis was a significant predictor for postoperative weight gain ≥5% [OR 0.97, 95% CI (0.94–1.00), P = 0.023] after adjusting for gender and follow-up time (model 1). However, this association disappeared [OR 0.98, 95% CI (0.95–1.01), P = 0.181] when additionally adjusting for preoperative BMI and radiotherapy (model 2). Preoperative BMI remained a significant risk factor [OR 0.81, 95% CI (0.70–0.93), P = 0.003] for postoperative weight gain ≥5% even after adjusting for age at diagnosis, gender, follow-up time, radiotherapy, pathologic subtype, and preoperative CDI (model 3). In model 3, pathologic subtype and preoperative CDI were also independent indicators of postoperative weight gain ≥5%. The adamantinomatous subtype conferred a higher risk compared with the papillary variant [OR 4.58, 95% CI (1.40–14.91), P = 0.012]. In addition, patients with preoperative CDI had a higher risk compared with the control [OR 3.09, 95% CI (1.02–9.37), P = 0.047]. After further adjustment by preoperative hypothalamus involvement (model 4), preoperative BMI [OR 0.78, 95% CI (0.67–0.90), P = 0.001] and pathologic subtype [adamantinomatous *vs*. papillary subtype, OR 3.46, 95% CI (1.02–11.76), P = 0.047] remained as significant risk factors for postoperative weight gain ≥5%; however, preoperative CDI was no longer significantly associated with postoperative weight gain ≥ 5%.

**Table 5:** Risk factors for postoperative weight gain **≥5%**.

Variables	Unadjusted OR (95% CI)	P value	Model 1 OR (95% CI)	P value	Model 2 OR (95% CI)	P value	Model 3 OR (95% CI)	P value	Model 4 OR (95% CI)	P value
Age at diagnosis	0.97 (0.94-1.00)	0.022	0.97 (0.94-1.00)	0.023	0.98 (0.95-1.01)	0.181	0.98 (0.95-1.01)	0.241	0.98 (0.94-1.02)	0.265
Preoperative BMI	0.80 (0.70-0.90)	<0.001			0.80 (0.71-0.91)	0.001	0.81 (0.70-0.93)	0.003	0.78 (0.67-0.90)	0.001
Radiotherapy										
No^*^	1				1		1		1	
Yes	0.19 (0.04-0.88)	0.034			0.21 (0.04-1.09)	0.063	0.12 (0.01-1.11)	0.062	0.15 (0.02-1.47)	0.104
Pathologic subtype										
Papillary variant^*^	1						1		1	
Adamantinomatous variant	2.34 (0.95-5.78)	0.065					4.58 (1.40-14.91)	0.012	3.46 (1.02-11.76)	0.047
Preoperative CDI										
No^*^	1						1		1	
Yes	2.00 (0.89-4.46)	0.092					3.09 (1.02-9.37)	0.047	2.84 (0.88-9.21)	0.082

Model 1: age at diagnosis, gender, follow-up time.

Model 2: Model 1 + preoperative BMI, Radiotherapy.

Model 3: Model 2 + pathologic subtype, preoperative CDI.

Model 4: Model 3 + preoperative hypothalamus involvement. *Reference.

### Risk Factors for Postoperative HO

In our study, the prevalence of HO increased from 19.2% preoperatively to 29.2% at last follow-up after surgery. To identify risk factors for postoperative HO, we first compared preoperative clinical characteristics between patients with or without postoperative HO. As shown in **Table 1**, the mean age at onset (HO 39.22 ± 12.59 *vs*. non-HO 41.47 ± 14.30, P = 0.421) and at diagnosis (HO 40.57 ± 12.43 *vs*. non-HO 43.40 ± 14.13, P = 0.305) was comparable between the two groups. No differences were found in the proportion of males (68.6 *vs*. 54.1% in HO and non-HO groups, respectively, p = 0.144), follow-up time [HO 12.0 (6.0–16.0) *vs*. non-HO 12.0 (4.0–13.0), months, P = 0.115] and the prevalence of intracranial hypertension symptoms (57.1 *vs*. 44.7% in HO and non-HO groups, p = 0.215) between the two groups. Notably, the HO group already had higher body weight (80.77 ± 14.24 *vs*. 63.81 ± 9.97, P < 0.001) and BMI (28.60 ± 4.16 *vs*. 22.81 ± 2.68, P < 0.001) preoperatively. There were no significant differences in tumor size and consistency between groups. But the ratio of grade 2 hypothalamus involvement was much higher in the HO group than in the non-HO group preoperatively (71.4 *vs*. 34.1%, P = 0.001). The treatment parameters, pathologic subtypes, rate of tumor relapse, and image-based postoperative hypothalamus involvement were similar between groups. Both prevalence of preoperative (**Table 2**) and postoperative endocrinopathy (**Table 3**) were comparable between the two groups, except that the HO group tended to have higher IGF-1 preoperatively [146.5 (113.50–187.75) *vs*. 124.50 (85.73–169.50)μg/L, P = 0.081] and a higher percentage of postoperative CDI (81.7 *vs*. 77.6%, P = 0.076), although not statistically significant.

**Figure 4:**
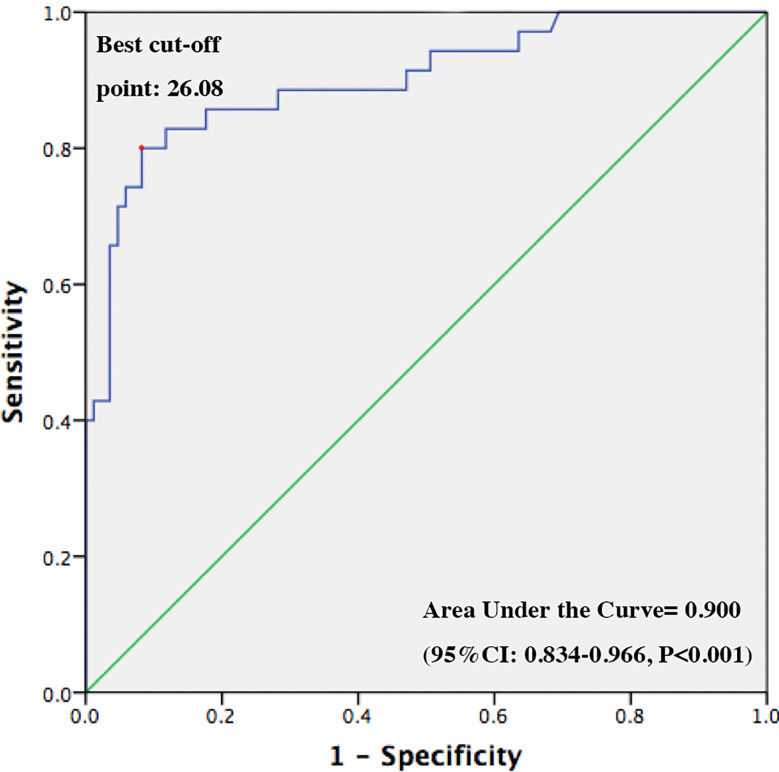
ROC curve for predicting postoperative hypothalamic obesity using preoperative BMI.

Furthermore, we conducted multivariable logistic regression analysis to identify independent risk factors for postoperative HO. As shown in **Table 6**, after adjusting for age at diagnosis, gender and follow-up time (model 5), patients with grade 2 hypothalamus involvement conferred a higher risk for developing HO postoperatively compared with patients without hypothalamus involvement (grade 0) preoperatively [OR 3.53, 95% CI (1.03–12.12), P = 0.045). This association disappeared when further adjusting for preoperative IGF-1, postoperative CDI and preoperative BMI (model 6). By contrast, preoperative grade 1 hypothalamus involvement [OR 0.12, 95% CI (0.02–0.98), P = 0.048] turned to be significantly associated with postoperative HO in model 6. Preoperative serum IGF-1 [OR 1.01, 95% CI (1.00–1.03), P = 0.040] and BMI [OR 2.25, 95% CI (1.60–3.16), P < 0.001] were also identified as independent risk factors for postoperative HO in this model. We next added pathologic subtype and radiotherapy into model 6 and found only preoperative BMI remained a significant risk factor for postoperative HO (model 7). Lastly, ROC curve was established to find out the optimal cut-off value of preoperative BMI to predict postoperative HO (**Figure 4**). It turned out that preoperative BMI >26.08 kg/m^2^ has the maximal sensitivity (0.8) and specificity (0.918) (area under the curve 0.900; 95% confidence interval 0.834–0.966, P < 0.001).

**Table 6:** Risk factors for postoperative Hypothalamic Obesity.

Variables	Unadjusted OR (95% CI)	P value	Model 5 OR (95% CI)	P value	Model 6 OR (95% CI)	P value	Model 7 OR (95% CI)	P value
Preoperative hypothalamus involvement								
Grade 0^*^	1		1		1		1	
Grade 1	0.65 (0.16-2.62)	0.548	0.68 (0.17-2.74)	0.582	0.12 (0.02-0.98)	0.048	0.08 (0.01-1.00)	0.050
Grade 2	3.66 (1.09-12.33)	0.036	3.53 (1.03-12.12)	0.045	0.69 (0.11-4.22)	0.686	0.31 (0.04-2.65)	0.282
Preoperative IGF-1	1.01 (1.00-1.01)	0.055			1.01 (1.00-1.03)	0.040	1.01 (1.00-1.03)	0.071
Postoperative CDI	3.07 (0.85-11.14)	0.088			9.28 (0.84-102.55)	0.069	15.93 (0.87-292.73)	0.062
Preoperative BMI	1.91 (1.50-2.44)	<0.001			2.25 (1.60-3.16)	<0.001	2.51 (1.64-3.85)	<0.001

Model 5: age at diagnosis, gender, follow-up time, preoperative hypothalamus involvement.

Model 6: Model 5 + preoperative IGF-1, postoperative CDI, preoperative BMI.

Model 7: Model 6 + pathologic subtype, radiotherapy.

*Reference.

Since the WHO criterion for obesity is BMI ≥30.0 kg/m^2^, we performed additional analyses based on this criterion. As shown in **Supplementary Table S2**, 20 (16.7%) patients developed HO postoperatively. Univariate analysis of clinical characteristics between patients with and without postoperative HO showed similar results as the above analysis, except that there was a higher rate of male (85.0 *vs*. 53.0% in the HO and non-HO groups, respectively, p = 0.008) and longer follow-up time (HO 13.0 (7.3–20.5) *vs*. non-HO 12.0 (4.0–13.0), months, P = 0.041) in the HO group. Multivariable logistic regression analyses (**Supplemental Table S3**) showed that male patients [OR 5.80, 95% CI (1.51–22.23), P = 0.001] conferred a higher risk for postoperative OB after adjusting for age at diagnosis and follow-up time (model 8). However, this association disappeared after further adjusting for preoperative hypothalamus involvement and preoperative BMI (model 9). Finally, preoperative BMI remained as a significant risk factor [OR 1.43, 95% CI (1.16–1.78), P = 0.001] for postoperative HO even when further adjusting for pathologic subtype and radiotherapy (model 10).

## Discussion

HO has been reported as a troublesome complication of craniopharyngioma. It increases risk for metabolic and cardiovascular diseases, resulting in impaired quality of life and excess mortality (5, 6). However, only a few studies were conducted in adult-onset craniopharyngioma patients. In the present study, we demonstrated a significant increase of body weight and BMI after surgery in patients with adult-onset craniopharyngioma in a median follow-up time of 12.0 months. Forty point eight percent of patients had clinically meaningful weight gain (≥5%) after surgery. In addition, we found lower preoperative BMI and adamantinomatous subtype were independent risk factors for postoperative weight gain ≥5%. The prevalence of HO was 19.2% preoperatively and increased to 29.2% at last follow-up postoperatively. After adjusting for age, gender, follow-up time, preoperative image-based hypothalamus involvement, pituitary function, pathologic subtypes, and radiotherapy, only preoperative BMI was found to be an independent risk factor for postoperative HO. Furthermore, we identified preoperative BMI >26.08 kg/m^2^ as the optimal cut-off value to predict postoperative HO.

The hypothalamus has long been recognized as the core of body weight regulation. The ventromedial nucleus (VMN) was once regarded as “satiety area”. Furthermore, the arcuate nucleus (ARC) has been found a major integration site for peripheral signals, such as leptin, insulin, and ghrelin. It can also project to the paraventricular nucleus (PVN), the lateral hypothalamus (LH), and other regions of the central nervous system, working together for energy balance (18). All of these hypothalamus nuclei are prone to damage caused by expansion of suprasellar tumors. Animal studies from experimentally induced combined medial hypothalamic lesions to rats demonstrated obesity phenotypes mimicking that of patients suffering from weight gain after treatment of craniopharyngioma (19, 20). Interruption of signal transduction of anorectic hormones, such as leptin and insulin in the hypothalamus is one probable mechanism leading to postoperative weight gain (21, 22). Another mechanism involves autonomic disturbances, that is, increased parasympathetic and reduced sympathetic tone (23, 24).

Here, we showed the prevalence of HO in our cohort was much higher than the recently reported prevalence of obesity in Chinese adults (16.4%) (25), especially postoperatively. This finding indicated that obesity was one of the major complications in adult-onset craniopharyngioma patients. This is consistent with previous findings that obesity was more prevalent in adult-onset craniopharyngioma patients than in the general population (26–28). It is noteworthy that the rate of HO in our cohort (19.2% preoperatively and 29.2% at last follow-up) was rather lower than that reported in recent studies in the USA or Europe (33.0–38.0% preoperatively and 46.0–55.6% at last follow-up) (26–28). Considering the prevalence of obesity in general population in China (16.4%) (25) was also much lower than that in the USA (42.4%) (29) or Europe (18–30%) (30), we favor the interpretation that the differences in the prevalence of HO between our study and others were at least partially due to ethnical differences. In a previous series with mixed childhood-onset and adult-onset craniopharyngioma patients in China, the incidence of HO in adult patients was reported to be 55.5% (31). However, the criterion adopted for obesity was BMI >25 kg/m^2^ in that study, which should be the criterion for overweight according to the standard of WHO. Thus, the rate of HO was obviously overestimated in that study. Multicenter prospective studies were needed to further investigate the prevalence of HO in adult craniopharyngioma patients in China.

Another important finding from our study is that nearly half of patients developed clinically meaningful weigh gain after surgery. With available data at each follow-up time points, we demonstrated that body weight and BMI kept increasing within the first year following surgery. This finding is consistent with previous studies conducted in childhood craniopharyngioma patients that significant increase in BMI occurred mainly during the first 6 months to 3 years after therapy, then the BMI sustained at follow-up (32, 33). In order to unravel the pattern of weight gain in adult-onset craniopharyngioma patients after 1 year following resection, further studies with longer follow-up time are needed.

Moreover, adamantinematous subtype was identified as an independent risk factor for postoperative weight gain. This is reminiscent of a recent study carried out in adult craniopharyngioma patients showing that both preoperative and postoperative obesity rates tended to be lower in papillary craniopharyngioma patients than that in adamantinematous patients, though statistical significance was not reached (27). It is well known that these two kinds of craniopharyngioma have different genetic mutations, clinical and image characteristics, including incidence, age of onset, tumor origin, and invasion pattern, *etc.* (34). Whether pathologic subtypes contribute to the development of HO in adult craniopharyngioma patients needs to be further explored.

Interestingly, we found that patients with higher preoperative BMI gained less weight following surgery, which was also observed by Duan et al. in their adult-onset craniopharyngioma patients (28). However, higher preoperative BMI was also an independent risk factor for postoperative HO, which was supported by several previous studies conducted in child-onset craniopharyngioma patients (32, 35, 36). In another word, those patients with preoperative HO often kept obese after surgery, without further weight gain. One possible explanation is that patients who developed HO preoperatively had their hypothalamus structure damaged by the tumor itself, and a plateau in weight gain was achieved. Supporting this explanation is the finding that patients with preoperative HO has a significant higher rate of hypothalamus involved in preoperative MRI (data not shown).

Although damage to the hypothalamus caused by the tumor or resection was the generally accepted cause of HO, image-based hypothalamus involvement was not identified as an independent risk factor for postoperative weight gain or HO in our cohort. Previous studies that investigated the relationship between radiographic hypothalamic involvements and HO showed confounding results. Van Gompel et al. (13) reported a positive correlation between MRI-graded hypothalamus involvement and postoperative weight gains in a relatively small cohort of adult craniopharyngioma patients. Similarly, a higher prevalence of obesity was described in patients with grade 2 hypothalamus involvement in a mixed cohort of child-onset and adult-onset craniopharyngioma patients (37). However, multivariable logistic regression adjusting for cofounders was not performed in these two studies; thus, it is hard to draw any conclusion in the relationship between image-based hypothalamic involvement and HO from these studies. On the contrary, several studies (27, 28, 38) failed to detect any correlation between neuroimaging features and postoperative weight gain or obesity, consistent with our findings. One possible explanation for this was that the MRI grading systems used in the above studies to assess hypothalamus involvement might be misleading and could not reflect the true status of hypothalamus. New MRI scoring system (39) and classification systems based on tumor origin or growth pattern (40, 41) were reported in recent years, each of which showed good promise in predicting hypothalamus damage and postoperative weight gain or obesity. Large-scale clinical studies are needed to further evaluate the validation of these new classification systems.

We found no relation between any endocrinopathies and postoperative weight gain or HO either. In previous studies, inconsistent results were reported in the relationship between endocrine deficiencies and HO (12, 27, 28, 35). Here in this study, pituitary function has been assessed at each follow-up, and dosages of substitutive drugs were adjusted accordingly. We therefore speculate proper substitution therapy might in some extent disguise the effect of endocrine deficiencies in postoperative weight gain. However, growth hormone deficiency, which might be a complicating factor of weight gain, was not evaluated regularly in the study population and no subjects had received growth hormone replacement. Although growth hormone replacement therapy in growth hormone deficient adult patients is beneficial to body composition, metabolic characteristics, quality of life, *etc.*, several long-term studies failed to show consistent effects on body weight and BMI (42–44). Moreover, total body weight and BMI seem to change little with GH treatment in adults (43). Further prospective studies, therefore, are needed to determine whether growth hormone deficiency is a major determinant of postoperative weight gain in adult-onset craniopharyngioma patients.

Our study had some limitations. The retrospective study design prevented assessing diet and exercise along with postoperative weight change. Secondly, only patients with available follow-up data were analyzed. It is possible that those with postoperative weight gain or other discomforts were more likely to be admitted to hospital, which might have caused overestimation of all complications. Thirdly, the limited follow-up duration might affect the validities of our findings on postoperative weight gain.

Based on our results and those in the literature, it is advisable to have objective risk assessments for postoperative weight gain in adult-onset craniopharyngioma patients so that individual treatment algorithm with hypothalamus sparing to the greatest possible extent for those at higher risks for developing postoperative HO can be instituted. Close follow-up and preventive measures, such as diet, behavior modification, and medical interventions can be employed early. These data also highlight the need for large and prospective studies of weight changes in adult-onset craniopharyngioma patients as well as controlled intervention trials in this specific population.

## Conclusions

The results from the present study suggest the following: 1) like in pediatric craniopharyngioma patients, postoperative weight gain and HO are important morbidities in adult-onset patients; 2) weight gain occurs throughout the first year following surgery; 3) lower preoperative BMI, and adamantinematous subtype predicts postoperative weight gain; 4) preoperative BMI predicts postoperative HO.

## Data Availability Statement

The data analyzed in this study is subject to the following licenses/restrictions: The Data may be related to the privacy of clinical subjects. Requests to access these datasets should be directed to wuwei12@fudan.edu.cn.

## Ethics Statement

The studies involving human participants were reviewed and approved by Institutional review board of Huashan Hospital, Fudan University. The patients/participants provided their written informed consent to participate in this study.

## Author Contributions

WW, YW, YL, and HY conceived the project. YW performed most of the surgery of patients in this study. YL, HY, QM, and QZ performed preoperative and postoperative endocrine examinations of all the subjects. WW, QS, and BX collected data. WW, XZ, and BX analyzed data. WW, QS, XZ, and HY wrote the manuscript. All authors contributed to the article and approved the submitted version.

## Funding

This work was supported by the National Key Research and development program of China (2019YFA0801900), National Nature Science Foundation for Young Scientists of China (No. 82000790) and Initial Scientific Research Fund of Huashan Hospital North (No. HSBY2017009).

## Conflict of Interest

The authors declare that the research was conducted in the absence of any commercial or financial relationships that could be construed as a potential conflict of interest.

## Publisher’s Note

All claims expressed in this article are solely those of the authors and do not necessarily represent those of their affiliated organizations, or those of the publisher, the editors and the reviewers. Any product that may be evaluated in this article, or claim that may be made by its manufacturer, is not guaranteed or endorsed by the publisher.
