# Supplementary material for: Risk Factors for Hypothalamic Obesity in Patients With Adult-Onset Craniopharyngioma: A Consecutive Series of 120 Cases
Source: Front Endocrinol (Lausanne). 2021 Jul 28;12:694213. doi: 10.3389/fendo.2021.694213 (PMC8355989; doi:10.3389/fendo.2021.694213)
Supplement: Supplementary file 1 [file DataSheet_1.docx]

Supplemental Table 1. Predictive accuracies and P values of tests for goodness of fit of multivariable logistic regression analyses

| Model | Predictive accuracy | P value for HL test |
| --- | --- | --- |
| Model 1 | 63.3% | 0.675 |
| Model 2 | 69.2% | 0.638 |
| Model 3 | 73.3% | 0.640 |
| Model 4 | 71.3% | 0.313 |
| Model 5 | 70.8% | 0.931 |
| Model 6 | 89.7% | 0.832 |
| Model 7 | 91.1% | 0.845 |
| Model 8 | 84.2% | 0.673 |
| Model 9 | 88.3% | 0.657 |
| Model 10 | 88.1% | 0.525 |

Supplemental Table 2. Clinical Characteristics and pituitary functions of 120 adult patients with craniopharyngioma categorized by postoperative HO defined as BMI ≥30.0kg/m^2^.

| Variables | Postoperative HO group  (N=20) | Postoperative non-HO group  (N=100) | P value |
| --- | --- | --- | --- |
| Age at onset, years | 36.60±13.32 | 41.66±13.81 | 0.135 |
| Age at diagnosis, years | 37.85±13.14 | 43.52±13.64 | 0.090 |
| Gender, male (%) | 17 (85.0%) | 53 (53.0%) | 0.008 |
| Follow-up time, months | 13.0 (7.3-20.5) | 12.0 (4.0-13.0) | 0.041 |
| Intracranial hypertension symptoms | 12 (60.0%) | 46 (46.0%) | 0.253 |
| Preoperative body weight, Kg | 84.22±16.16 | 65.66±10.86 | <0.001 |
| Preoperative BMI, Kg/m^2^ | 29.02±5.22 | 23.59±3.20 | <0.001 |
| Body weight at last follow-up, Kg | 94.54±9.13 | 68.33±10.19 | <0.001 |
| BMI at last follow-up, Kg/m^2^ | 32.60±2.13 | 24.56±2.91 | <0.001 |
| Image characteristics |  |  |  |
| Tumor size, mm | 31.83±8.10 | 29.78±9.77 | 0.393 |
| Tumor consistency |  |  | 0.632 |
| Predominantly cystic, n (%) | 6 (30.0%) | 32 (32.0%) |  |
| Predominantly solid, n (%) | 3 (15.0%) | 24 (24.0%) |  |
| Mixed, n (%) | 11 (55.0%) | 44 (44.0%) |  |
| Preoperative hypothalamus involvement, n (%) |  |  | 0.013 |
| Grade 0 | 1 (5.0%) | 20 (20.0%) |  |
| Grade 1 | 4 (20.0%) | 41 (41.0%) |  |
| Grade 2 | 15 (75.0%) | 39 (39.0%) |  |
| Postoperative hypothalamus involvement, n (%) |  |  | 0.332 |
| Grade 0 | 6 (30.0%) | 40 (40.0%) |  |
| Grade 1 | 6 (30.0%) | 36 (36.0%) |  |
| Grade 2 | 8 (40.0%) | 24 (24.0%) |  |
| Treatment parameters |  |  |  |
| Surgical approach |  |  | 1.000 |
| Endoscopic Endonasal Approach | 19 (95.0%) | 94 (94.0%) |  |
| Transcranial approach | 1 (5.0%) | 6 (6.0%) |  |
| Extent of surgery |  |  | 0.614 |
| Gross total resection | 16 (80.0%) | 68 (68.0%) |  |
| Subtotal resection | 4 (20.0%) | 26 (26.0%) |  |
| Partial resection | 0 (0.0%) | 6 (6.0%) |  |
| Radiotherapy, n (%) | 1 (5.0%) | 14 (14.0%) | 0.459 |
| Pathologic subtype^*^ |  |  | 0.968 |
| Adamantinomatous variant, n (%) | 11 (68.8%) | 58 (68.2%) |  |
| Papillary variant, n (%) | 5 (31.3%) | 27 (31.8%) |  |
| Tumor relapse, n (%) | 5 (25.0%) | 24(24.0%) | 1.000 |
| Preoperative pituitary function |  |  |  |
| Adrenal insufficiency | 7 (35.0%) | 36 (36.0%) | 0.932 |
| Hypothyroidism | 7 (35.0%) | 47 (47.0%) | 0.325 |
| Hypogonadism | 15 (75.0%) | 78 (78.0%) | 1.000 |
| Central diabetes insipidus | 8 (40.0%) | 26 (26.0%) | 0.205 |
| Number of pituitary deficits | 1.5 (1.0-3.0) | 2.0 (1.0-3.0) | 0.977 |
| 4 pituitary deficits |  |  |  |
| IGF-1 (μg/L) | 138.0 (109.0-181.0) | 130.0 (97.0-174.0) | 0.612 |
| IGF-1 below the reference range | 7 (35.0%) | 38 (38.0%) | 0.800 |
| Postoperative pituitary function |  |  |  |
| Adrenal insufficiency | 19 (95.0%) | 83 (83.0%) | 0.303 |
| Hypothyroidism | 18 (90.0%) | 87 (87.0%) | 1.000 |
| Hypogonadism | 19 (95.0%) | 89 (89.0%) | 0.683 |
| Central diabetes insipidus | 18 (90.0%) | 80 (80.0%) | 0.460 |
| Number of pituitary deficits | 4.0 (4.0-4.0) | 4.0 (3.0-4.0) | 0.225 |
| 4 pituitary deficits |  |  |  |
| IGF-1 (μg/L) | 92.3 (64.4-106.5) | 91.0 (62.1-131.0) | 0.696 |
| IGF-1 below the reference range | 19 (95.0%) | 83 (83.0%) | 0.303 |

Supplemental Table 3. Risk factors for postoperative HO defined as BMI ≥30.0kg/m^2^.

| Variables | Unadjusted  OR (95% CI) | P value | Model 8  OR (95% CI) | P value | Model 9  OR (95% CI) | P value | Model 10  OR (95% CI) | P value |
| --- | --- | --- | --- | --- | --- | --- | --- | --- |
| Age at diagnosis | 0.97 (0.93-1.01) | 0.094 | 0.96 (0.92-1.00) | 0.065 | 0.95 (0.90-1.01) | 0.092 | 0.96 (0.91-1.02) | 0.182 |
| Gender |  |  |  |  |  |  |  |  |
| Female^*^ | 1 |  | 1 |  | 1 |  | 1 |  |
| Male | 5.02 (1.39-18.23) | 0.014 | 5.80 (1.51-22.23) | 0.010 | 3.76 (0.87-16.31) | 0.077 | 2.98 (0.60-14.83) | 0.182 |
| Follow-up time | 1.06 (1.00-1.12) | 0.052 | 1.06 (1.00-1.12) | 0.064 | 1.06 (0.98-1.15) | 0.151 | 1.08 (0.95-1.23) | 0.230 |
| Preoperative hypothalamus involvement |  |  |  |  |  |  |  |  |
| Grade 0^*^ | 1 |  |  |  | 1 |  | 1 |  |
| Grade 1 | 1.95 (0.21-18.62) | 0.561 |  |  | 1.49 (0.13-17.20) | 0.749 | 1.25 (0.09-18.34) | 0.872 |
| Grade 2 | 7.69 (0.95-62.49) | 0.056 |  |  | 3.12 (0.32-30.27) | 0.326 | 1.77 (0.15-20.58) | 0.649 |
| Preoperative BMI | 1.46 (1.22-1.74) | <0.001 |  |  | 1.41 (1.17-1.71) | <0.001 | 1.43 (1.16-1.78) | 0.001 |

Model 8: age at diagnosis, gender, follow-up time.

Model 9: Model 8 + preoperative hypothalamus involvement, preoperative BMI.

Model 10: Model 9 + pathologic subtype, radiotherapy.

^*^ Reference
